# Reassessing Fano Resonance for Broadband, High‐Efficiency, and Ultrafast Terahertz Wave Switching

**DOI:** 10.1002/advs.202204494

**Published:** 2022-11-17

**Authors:** Yuze Hu, Mingyu Tong, Siyang Hu, Weibao He, Xiang'ai Cheng, Tian Jiang

**Affiliations:** ^1^ Institute for Quantum Science and Technology College of Science National University of Defense Technology Changsha 410073 P. R. China; ^2^ College of Advanced Interdisciplinary Studies National University of Defense Technology Changsha 410073 P. R. China

**Keywords:** Fano resonance, near‐field coupling, reconfigurable metasurfaces, terahertz switching, ultrafast photonics

## Abstract

Miniaturized ultrafast switchable optical components with high efficiency and broadband response are in high demand to the development of optical imaging, sensing, and high‐speed communication. Sharp Fano‐type resonance switched by active materials is one of the key concepts that underpins the control of light in metaoptics with high sensitivity. However, actuating such metasurfaces exhibits a long‐standing trade‐off between modulation depth and operational bandwidth. Here, the limitations are circumvented by theoretical analysis, numerical simulation, and experimental realization of an achromatic Fano metasurface so that a high contrast of tunability with ultrafast switching rate over a broad range of frequency is achieved. By developing the physics of inter‐mode coupling, the Fano metasurface is designed according to a complete phase diagram derived from coupled mode theory. Unlike conventional Fano metasurfaces, the cross‐polarized inter‐metaatoms coupling is discovered as a superior ability of high‐efficiency broadband achromatic polarization conversion. To prove the ultrasensitive nature, a metadevice is constructed by incorporating a thin amorphous Ge layer with a weak photoconductivity perturbation. Transmission modulation over broadband frequency range from 0.6 to 1.1 THz is thus successfully realized, featuring its merits of modulation depth over 90% and On–Off–On switching cycle less than 10 ps.

## Graphical abstract

Fano‐type metamaterials switched by active materials enable the control of light in metaoptics with high sensitivity. However, they exhibit a long‐standing trade‐off between modulation depth and operational bandwidth. Here, the authors circumvent this limitation by proposing a novel Fano‐type metasurface with high contrast of tunability and broadband ultrafast switching, which is extremely appealing for next‐generation flat photonic devices.

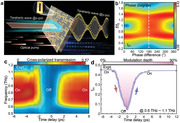

## 1 Introduction

Between microwave and optical waves, terahertz (THz) electromagnetic radiation occupies a frequency range of 0.1 to 10 THz.^[^ ^1^ ^]^ By combining the advantages of two neighboring spectral bands, THz radiation holds an extremely appealing prospect for technological innovation in security verification,^[^ ^2^ ^]^ high‐capacity communications,^[^ ^3^, ^4^ ^]^ imaging,^[^ ^5^ ^]^ spectroscopy,^[^ ^6^, ^7^ ^]^ and biosensing.^[^ ^8^ ^]^ However, owing to the lack of relevant functional devices in the THz regime, it is also known as “THz gap”. Instead of conventional integrated circuits, which are progressively nearing the Moore's Law bottleneck, the heart of next‐generation all‐optical systems will be miniaturized ultrafast switchable optical components with an incredibly tiny size and higher service. Functional reconfigurable devices featuring high response rates have drawn a lot of interest recently as a result of the rising demand for ultra‐high‐speed applications for next‐generation communication.^[^ ^9^, ^10^, ^11^ ^]^ The metamaterials integrated with active elements provide a new paradigm of dynamically manipulating the light–matter interactions over the near‐fields, greatly meriting their applications in far‐field wave manipulations.^[^ ^12^, ^13^ ^]^ There is no debate about the significance of active, adjustable, or reconfigurable components in contemporary electromagnetic and photonic systems.^[^ ^14^, ^15^ ^]^ Due to its adjustable and on‐demand optical qualities, THz microcavities in the form of artificially constructed subwavelength metamaterials have received considerable attention in the THz photonics field.^[^ ^16^, ^17^, ^18^, ^19^ ^]^ Recent developments in functional THz metadevices have drawn attention toward versatile modulations by using mechanical,^[^ ^20^, ^21^, ^22^, ^23^ ^]^ electrical,^[^ ^24^, ^25^ ^]^ thermal,^[^ ^26^, ^27^ ^]^ magnetic,^[^ ^28^ ^]^ and optical^[^ ^29^, ^30^ ^]^ approaches. To fully exploit the potential of terahertz radiation, the urgent application‐side demands have propelled this topic ahead swiftly and elevated active metadevices to be the frontier of metasurface research.^[^ ^31^, ^32^ ^]^

Recent research has revealed the existence of the Fano‐type resonance in electromagnetic metamaterials, photonic crystals, and plasmonic nanoparticles.^[^ ^33^, ^34^ ^]^ Fano resonance is a hybridized mode leading to a narrowband discrete state interfering from a broadband continuum when two or more nanostructures are placed next to each.^[^ ^35^, ^36^ ^]^ This lineshape is ubiquitous from various photonic phenomenon, such as electromagnetically induced transparency,^[^ ^37^ ^]^ parity‐time symmetry breaking,^[^ ^38^ ^]^ quasi‐bound states in the continuum,^[^ ^39^, ^40^ ^]^ and topological edge state.^[^ ^41^, ^42^ ^]^ The steep dispersion of the Fano resonance profile,^[^ ^43^, ^44^ ^]^ in contrast to standard symmetric resonating curves, promises a variety of applications in sensors,^[^ ^45^, ^46^ ^]^ lasing,^[^ ^47^, ^48^ ^]^ switching, nonlinear,^[^ ^49^ ^]^ and slow‐light devices.^[^ ^50^, ^51^ ^]^ These unique features naturally motivate further investigations on ultracompact terahertz modulators. There have been several attempts to implement active Fano systems utilizing different external stimuli. For instance, Fano resonances have been changed by mechanically actuating the microelectromechanical system,^[^ ^52^, ^53^ ^]^ thermally tuning superconductor Ohmic loss^[^ ^54^ ^]^ or phase transition materials,^[^ ^55^, ^56^, ^57^ ^]^ electrically controlling graphene^[^ ^58^, ^59^, ^60^ ^]^ or liquid crystal.^[^ ^61^, ^62^ ^]^ More importantly, the unique sensitive feature of sharp Fano resonance has opened up a new chapter for ultrafast all‐optical switching by incorporating numerous photoactive materials, including intrinsic/ion‐planted silicon,^[^ ^63^, ^64^ ^]^ amorphous germanium,^[^ ^65^ ^]^ superconductors,^[^ ^66^ ^]^ perovskites,^[^ ^67^, ^68^, ^69^ ^]^ topological insulators,^[^ ^70^ ^]^ and transition metal dichalcogenides.^[^ ^71^, ^72^, ^73^ ^]^ The increased field concentration in tiny volume supported by Fano metamaterials tremendously boosts an enough high modulation depth for weak photoconductivity perturbation, especially for transient relaxation time down to picosecond.^[^ ^74^, ^75^ ^]^ Thus, the Fano lineshape with high quality (Q) factors can complete a full switching cycle up to a hundred of a gigahertz, representing the immense potentials for THz communication. However, they have poor device control due to their small working band and the Fano resonance requires a sudden shift in dispersion across a limited spectral range. Therefore, there is still plenty of room to further advance this area of research. It is essential to reassert the principles in constructing Fano‐type metastructures, which presents immense potential for broadband, ultrasensitive, and dynamical components.

In this work, we combine theoretical, numerical, and experimental efforts to address this issue by establishing an active metadevice for high‐efficiency and ultrafast broadband terahertz wave switching. As one typical Fano‐type resonance, the spectral feature of coupled resonances comes into a different situation once two orthogonal polarizations are fully taken into consideration. After obtaining a complete phase diagram derived from temporal coupled mode theory, we provide a general physical guidance for designing Fano metasurface with an achromatic property. The proposed concept guides us to experimentally realize a high‐performance reconfigurable metadevice. Transmission modulation in the broadband spectral range from 0.6 to 1.1 THz is successfully realized, featuring its merits of modulation depth over 90% and On–Off–On switching cycle less than 10 ps. As a weak photoconductivity perturbation is used in the experiments, the sensitive feature of Fano‐type resonances is also proved even though the sharp dispersion disappears. The discovery of achromatic polarization conversion effect not only develops the new physics of Fano‐type metasurfaces, but more importantly, it also lays a solid basis for functional and tunable metadevices.

## 2 Results

### 2.1 Establishment of a General Phase Diagram

To explain the necessity of developing novel Fano‐type metasurface, we would like to first point out the difficulties in achieving ultrafast broadband THz modulation with high‐efficiency using conversional resonances. To realize dynamic broadband tuning at picosecond timescale, the resonant metasurface must satisfy the following conditions. First, to induce significant transmission modulation from weak perturbation, the resonant mode of the metasurface must be Q‐factor enhanced by mode coupling so that its radiative loss rate is sensitive to the dissipative loss rate. Second, one should broaden the resonant frequency range, usually done by using a low‐Q resonator or by overlapping multiple resonances at near frequencies. Third, to maintain the uniform transmission amplitude over a broad spectral range, the hybrid mode must couple with the outgoing wave by sweeping the operating frequencies without obvious radiative loss rate variation. As outlined above, the following three objectives must be satisfied simultaneously for metasurface design: enhancing the Q‐factor by mode coupling, maximally broadening the working linewidth via multiple resonators, and minimally compressing the radiative loss rate change over a broadband frequency range. However, there lies an inherent trade‐off problem for accessing a broadband uniform transparency window if only a single polarization state is considered for the metaatom design.

**Figure 1:**
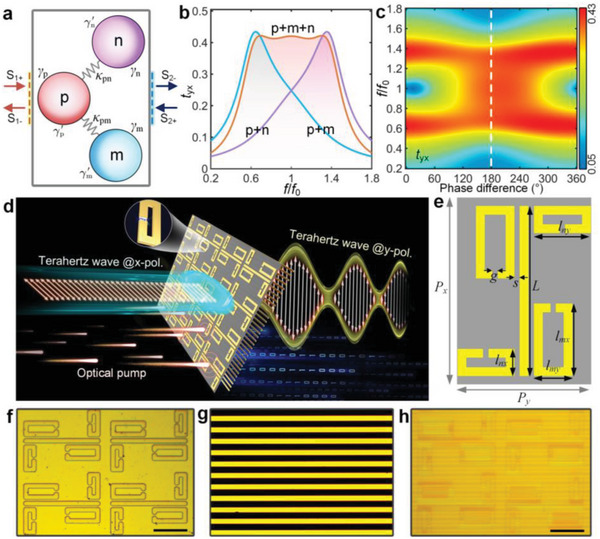
Design principle of the broadband tunable cross‐polarized Fano‐type metasurface. a) Schematic of a resonating system supporting one *x*‐polarized and two *y*‐polarized modes that are coupled to two ports. TCMT‐calculated transmission spectra of the proposed coupled modes under the influence of b) different combinations and c) varied coupling phases. d) Illustration of the cross‐polarization conversion effect for ultrafast photo‐switching broadband cross‐polarized THz wave at picosecond timescale. The metasurface is hybrid with a 200‐nm‐thick amorphous Ge to generate perturbations via photocarriers. e) Top‐view of the metasurface one‐unit cell. The key geometrical parameters are *L* = 120 µm, *l*_nx_ = 19 µm, *l*_ny_ = 41 µm, *l*_mx_ = 51 µm, *l*_mv_ = 27 µm, *s* = 4 µm, *g* = 4 µm, *P_x_* = 95 µm, and *P_v_* = 132 µm. f–h) Optical micrographs of the fabricated metasurface, metallic grating, and their combined structure, respectively.

Without the loss of generality, consider three coupling resonators exhibiting orthogonal eigenstates, which is described by the temporal coupled‐mode theory (TCMT) and schematized in **Figure** **1**a. Here, “p” is a resonator with *x*‐polarized eigenstate coupling with the incoming/outgoing wave, while “n” and “m” can only radiate the *y*‐polarized wave. In the language of ordinary electromagnetically induced transparency (EIT) effect by ignoring the *y*‐polarized waves, “p” can be regarded as the “bright” mode |1〉 and “m”/’n’ is the “dark” resonance |2〉 because it cannot be directly excited by *x*‐polarized incidence. Analogy to a three‐level EIT system in quantum context, the destructive interference between |0〉→|1〉 and |0〉→|1〉→|2〉→|1〉 would lead to the formation of transparency window in a narrow frequency range. Here, we theoretically extend the formula of an EIT‐like metasurface when the “dark” mode is radiative to the orthogonal polarization state. Assuming the Hamiltonian to describe the three eigenmodes in Figure 1a satisfies the mode eigenvalue equation $\hat{H}|\Psi_{i}\rangle =2\pi f_{i}\left|\Psi_{i}\right\rangle \left(i=px,my,ny\right)$; the wave function in the coupled system is |Ψ_*i*_〉 = ∑*a_i_* |Ψ_*i*_〉. Focusing on the solution of cross‐polarized transmission amplitude, our proposed metaatoms can thus be viewed as a three‐mode two‐port system. In addition to the radiative rate *γ* and coupling strength *κ*, the absorption decay rate *γ*′ of each resonator is taken into consideration to mimic active modulations. Taking polarization resolved incoming and outgoing waves at the 1st and 2nd ports $|s^{i}\rangle ={\left[s_{1x}^{i},s_{1y}^{i},s_{2x}^{i},s_{1y}^{i}\right]}^{T}\left(i=+,-\right)$ as an example, the TCMT model is defined as:^[^ ^76^, ^77^ ^]^

(1) $$\begin{array}{c} \begin{array}{c} \frac{1}{2\pi }\frac{\partial a}{\partial t}=-i\left[\left(\begin{array}{ccc} f_{px} & \;\kappa_{pxmy} & \;\kappa_{pxny}\\ \kappa_{mypx} & \;f_{my} & \;0\\ \kappa_{nypx} & \;0 & \;f_{ny} \end{array}\right)-i\left(\begin{array}{ccc} {\gamma^{\prime }}_{px} & \;0 & \;0\\ 0 & \;{\gamma^{\prime }}_{my} & \;0\\ 0 & \;0 & \;{\gamma^{\prime }}_{ny} \end{array}\right)\right]a\\ -\left(\begin{array}{ccc} \gamma_{px} & \;0 & \;0\\ 0 & \;\gamma_{my} & \;X_{myny}\\ 0 & \;X_{myny} & \;\gamma_{ny} \end{array}\right)a+\left(\begin{array}{cccc} d_{1px} & \;0 & \;d_{2px} & \;0\\ 0 & \;d_{1my} & \;0 & \;d_{2my}\\ 0 & \;d_{1ny} & \;0 & \;d_{2ny} \end{array}\right)|s^{+}\rangle \end{array} \end{array}$$

(2) $$|s^{-}\rangle \left(\begin{array}{cccc} r_{sub} & \;0 & \;t_{sub} & \;0\\ 0 & \;r_{sub} & \;0 & \;t_{sub}\\ t_{sub} & \;0 & \;r_{sub} & \;0\\ 0 & \;t_{sub} & \;0 & \;r_{sub} \end{array}\right)|s^{+}\rangle +\left(\begin{array}{ccc} d_{1px} & \;0 & \;0\\ 0 & \;d_{1my} & \;d_{1ny}\\ d_{2px} & \;0 & \;0\\ 0 & \;d_{2my} & \;d_{2ny} \end{array}\right)a$$

where **a** = [*a_px_*, *a_my_*, *a_ny_*]^T^ is a vector denoting the amplitude of three resonance modes: *κ*_ij_ (i,j = *b_x_*, *m_y_*, *n_y_*) describes the near‐field coupling between three modes and *κ*_ij_ and *κ*_ji_ are mutually conjugate numbers; *d*_ib_ *_x_* (*i* = 1,2) are the coupling coefficients between external light and the radiative modes at ports *i* = 1,2; and *r*_sub_ and *t*_sub_ constitute the background scattering matrix between the ports with the disappearance of resonance. It is worth stressing that the cross‐polarized transmission amplitude can thus be expressed as $t_{yx}=S_{2y}^{-}/S_{1x}^{+}$. To analytically solve the above equations, energy conservation and time‐reversal symmetry should be simultaneously satisfied:

(3) $$\begin{array}{c} \left(\begin{array}{cccc} r_{sub} & 0 & t_{sub} & 0\\ 0 & r_{sub} & 0 & t_{sub}\\ t_{sub} & 0 & r_{sub} & 0\\ 0 & t_{sub} & 0 & r_{sub} \end{array}\right)\;{\left(\begin{array}{ccc} d_{1px} & 0 & 0\\ 0 & d_{1my} & d_{1ny}\\ d_{2px} & 0 & 0\\ 0 & d_{2my} & d_{2ny} \end{array}\right)}^{*}\;=\;\left(\begin{array}{ccc} d_{1px} & 0 & 0\\ 0 & d_{1my} & d_{1ny}\\ d_{2px} & 0 & 0\\ 0 & d_{2my} & d_{2ny} \end{array}\right) \end{array}$$

(4) $$\begin{array}{c} \begin{array}{c} -\frac{1}{2}{\left(\begin{array}{ccc} d_{1px} & 0 & 0\\ 0 & d_{1my} & d_{1ny}\\ d_{2px} & 0 & 0\\ 0 & d_{2my} & d_{2ny} \end{array}\right)}^{†}\left(\begin{array}{ccc} d_{1px} & 0 & 0\\ 0 & d_{1my} & d_{1ny}\\ d_{2px} & 0 & 0\\ 0 & d_{2my} & d_{2ny} \end{array}\right)=-\left(\begin{array}{ccc} \gamma_{bx} & 0 & 0\\ 0 & \gamma_{my} & X_{myny}\\ 0 & X_{myny} & \gamma_{ny} \end{array}\right) \end{array} \end{array}$$

Along with standard CMT analysis, the cross‐polarized complex transmission coefficient is obtained from the joint solution of Equations (1)–(4),

(5) $$t_{\mathrm{yx}}=s_{2y}^{-}/s_{1x}^{+}=F\left(f_{i},\kappa_{\mathrm{ij}},\gamma_{i},\gamma_{i}^{\prime }\right)\left(i,j=b_{x},m_{y},n_{y}\right)$$

where *F* (*f*_i_, *κ*_ij_, *γ*_i_, *γ′*_i_) is a rather complicated expression which can be found in the Supporting Information, as shown in Equation S1, Supporting Information. As Equation (5) contains whole model parameters describing the underlying physics, we in principle receive a clear picture to guide the intriguing experimental behaviors reported in the next section. It is helpful to explicitly discuss intrinsic correlations between controllable lineshapes and conditions imposed on our coupling oscillators. By tuning the intrinsic parameters, Figure 1b,c depicts how to “design” uniform transmission amplitude with broadband feature by tuning the physical quantities. Here, we scale the resonant frequencies as *f_px_* = *f*_0_, *f_my,ny_* = *f_px_* ± 0.3*f*_0_. The radiative loss rates are set as *γ*_p_ *_x_* = 0.4*f*_0_ and *γ_my,ny_* = 0.08*f*_0_. The non‐radiative loss rates are *γ*_p_ *_x,my,ny_* = 0.01*f*_0_. The interresonator coupling amplitudes are |*κ*_p_ *_xmy_* | = |*κ*_p_ *_xny_* | = 0.22*f*_0_. Figure 1b unambiguously shows the influence of different combinations on the cross‐polarized lineshape when *κ*_p_ *_xmy_* = −*κ*_p_ *_xny_*. Consistent with our expectations, the coupling from two *x*‐polarized “dark” modes results in broadband *y*‐polarized transmission lineshape. Of particular interest is the efficient control of lineshape via relative phase difference between the resonances of “m” and “n”, as shown in Figure 1c. Increasing the phase difference between two resonators can drive *t_yx_* to change from discrete frequencies to a continuous frequency range, reaching an ideal shape when they are resonant out‐of‐phase.

### 2.2 Implement to Photonic Systems

**Figure 2:**
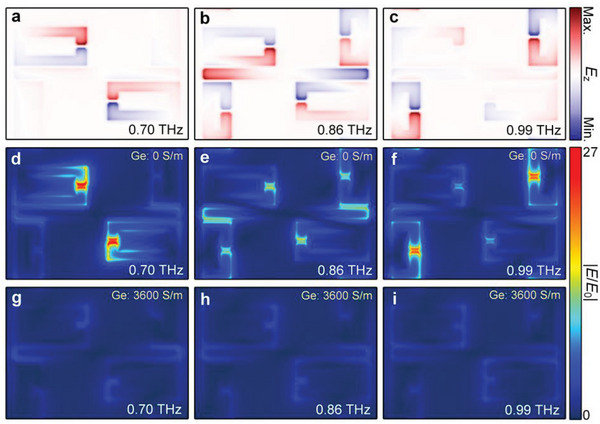
Electric field profiles for the cross‐polarized Fano‐type metasurface. a–c) The *z* component of *E*‐field distributions across the frequency range of the coupling induced broadband resonance. Calculated near‐field enhancement at various resonant frequencies d–f) without and g–i) with the perturbation of photoconductivity introduced by the 200‐nm‐thick Ge layer.

We now apply the above theoretical prediction to build the reconfigurable metadevice for high performance switching. Concept and design of the metasurface are graphically portrayed in Figure 1d. The cut‐wires (CWs) oriented along *x*‐direction act as a “bright” dipole moment corresponding to the “p” resonator, which can be excited by *x‐*polarized THz incidence. Two types of split ring resonators (SRRs) with different eigenfrequencies are distributed near the CW. The SRRs are excited by CWs with an inductance capacitance (LC) resonance only radiative to *y*‐polarized waves. It is noticeable that LC mode possesses a relatively high Q‐factor when compared with the electric dipole counterpart. As for the out‐of‐phase resonant condition between “m” and “n”, it is exceedingly necessary to arrange the SRRs with a special C_2_ symmetry as clearly seen from the top‐view in Figure 1e (detailed analysis will be carried out when discussing near‐field distributions of **Figure** **2**). After the construction of meta‐atoms, a 200‐nm‐thick amorphous Ge film is embedded into the gaps of SRRs through depositions. It serves as photon‐induced perturbations to greatly increase the non‐radiative loss of SRRs. In addition, a metal grating can be selectively used to block the co‐polarized waves and then further enhance the polarization conversion efficiency.^[^ ^78^ ^]^ The geometrical parameters of meta‐atoms are set as *L* = 120 µm, *l_nx_* = 19 µm, *l_ny_* = 41 µm, *l_mx_* = 51 µm, *l_mv_* = 27 µm, *s* = 4 µm, *g* = 4 µm, *P_x_* = 95 µm, and *P_y_* = 132 µm. The fabricated meta‐device is photographed by reflective optical microscopic, shown in Figure 1f–h, from which it is obvious that a Ge layer uniformly covers the metasurface and the metallic grating is located on the metasurface with the distance of 40 µm.

### 2.3 Theoretical Prediction of the Active THz Modulation

**Figure 3:**
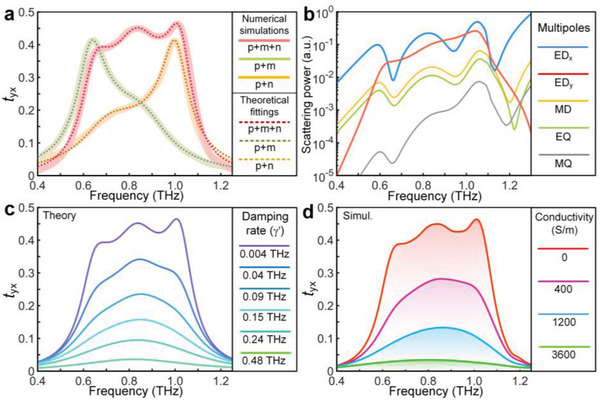
Theoretical interpretations of the proposed metadevice for broadband linear polarization conversion and efficient modulation. a) Cross‐polarized transmission of the single layer Fano metasurface and the corresponding fitted curves based on TCMT theory. b) Calculated far‐field scattering power of different multipoles: ED, electric dipole; MD, magnetic dipole; EQ, electric quadrupole; MQ, magnetic quadrupole. The scattering power of *y*‐polarized electric dipole is relatively high from 0.6 to 1.1 THz. c) TMCT calculated cross‐polarized transmission as a function of the non‐radiative damping rate. d) Numerical simulated cross‐polarized transmission under the perturbation of weak photoconductivity in a 200‐nm‐thick photoactive layer.

We now theoretically verify our predictions on active lineshape tailoring based on coupled systems constructed by the three kinds of resonators studied in Figure 1d. We perform finite element method (FEM) simulations (COMSOL Multiphysics) using the device and material parameters used in our experiments, which can predict the experimental results successfully. The underlying mechanism of efficient THz modulation is interpreted using the TCMT model by fitting the numerical spectra. **Figure** **3**a shows the cross‐polarized transmission spectra and their corresponding fitting curves with different resonance combinations. The elegant reproduction of simulation results in theoretical fittings verifies the correctness of the theoretical formula. It manifests that two high‐Q *y*‐polarized eigenmodes coupled with a low‐Q *x*‐polarized eigenmode could cause a broadband cross‐polarization conversion. To reveal the radiation patterns more clearly, a detailed multipole decomposition of the scattered fields is carried out. The far‐field scattering powers are plotted in Figure 3b, where one can see that the strongest contribution of the metasurface response is provided by the electric dipole (ED*_x_* and ED*_y_*). Notably, unlike the *x*‐polarized case, the *y*‐polarized electric dipole remains an overall high radiative power in the frequency ranging from 0.6 to 1.1 THz. This is one of the major merits of our proposed cross‐polarized Fano metasurface. We further perform an in‐depth investigation on the scattering powers and spectral responses with all combinations of resonators under either *x*‐ or *y*‐polarized THz incidence excitations. The numerical results in Figures S1 and S2, Supporting Information, clearly show that the *x*‐polarized “dark” SRRs can radiate *y*‐polarized electric dipoles across a broadband frequency. Another advantage of Fano resonance is the sensitivity to near‐field perturbations, which is highly useful in designing modulators or sensors.^[^ ^59^, ^64^, ^65^ ^]^ As presented in Figure 3c, the active annihilation of the broadband *y*‐polarized transmission arises from the change in the damping rate of the *x*‐polarized dark modes (“m” and “n”). The simulated data in Figure 3d yields the same modulation behavior by shorting of the capacitive split‐gap with a weak photoconductivity. Thus, it can be concluded from Figure 3 that an efficient broadband modulation is possible in our single‐layer cross‐polarized Fano metasurface, which offers a potential to overcome the limitation of ultrafast photo‐switching of a narrow frequency range. It is of interest to discuss the limited size of metasurface array. For typical plasmonic meta‐atoms, two kinds of resonances can be found, namely, localized resonance and surface lattice resonance. The latter is nonlocal and its resonant spectrum would be greatly influenced by the array size even though the array is larger than the light spot.^[^ ^79^ ^]^ Fortunately, the resonance modes of our structure are localized within one unit‐cell and the inter‐unit coupling is negligible. Thus, the spectrum would not vary a lot as long as the array size of the metasurface is larger than beam spot.

### 2.4 Experimental Verifications of Ultrafast Broadband THz Wave Tunability

**Figure 4:**
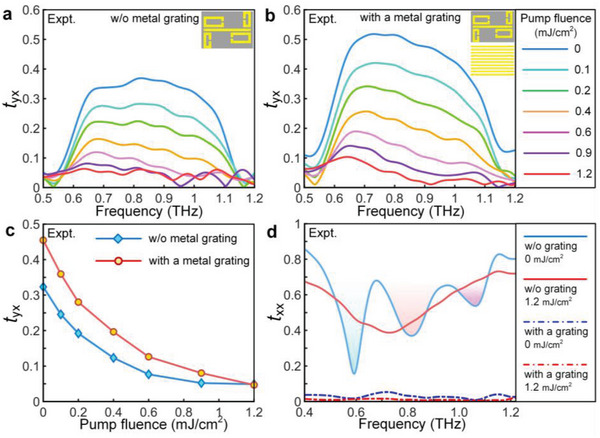
Experimental demonstration of the broadband THz wave tunability. The experimentally recorded photo‐switching of the cross‐polarized transmission spectra for the metasurface a) without metallic grating and b) with a metallic grating. c) Amplitude modulation of averaged cross‐polarized transmission ranging from 0.6 to 1.1 THz. d) Co‐polarized transmission spectra influenced by the photon injection and metallic grating, respectively.

In the following, we demonstrate applications of our metadevice to control the broadband transmission amplitude at picosecond timescale, which is important in terahertz spectroscopy and ultrafast photonics. We start with an experimental verification of the theoretical predictions via perturbations of photo‐carriers excited by femtosecond optical pulses. Owing to the time‐dependent switching behavior, the pump‐probe time delay of 0 ps is adopted when the maximal response occurs. The spectral modulation of the single layer metasurface is shown in **Figure** **4**a. It is visible that a uniform broadband cross‐polarized transmission takes place with an amplitude of 0.32 averaged from 0.6 to 1.1 THz. Nevertheless, the co‐polarized amplitude contains three obvious dips and two transparency windows in this frequency range, owing to the coupling between one bright mode with two dark modes. It indicates the observation of a typical EIT far‐field radiation feature for the co‐polarized component.^[^ ^80^ ^]^ Notably, the cross‐polarized component does not exhibit an EIT configuration but just a near‐field coupling of modes of different polarizations at different central frequencies.^[^ ^81^, ^82^ ^]^ More strikingly, the cross‐polarized transmission experiences a giant suppression as the pump fluence increases to 1.2 mJ cm^−2^, whereas the co‐polarized one just undergoes a slight amplitude change (red curves in Figure 4a,d). We next further improve the conversion efficiency and increase modulation depth by simply adding a metallic grating below the metasurface with 40‐µm distance. The co‐polarized transmission disappears (dotted curves in Figure 4d) and the corresponding cross‐polarized transmission controlling is monitored in Figure 4b. Without optical pump, the broadband averaged amplitude increases from 0.32 to 0.47, but it can also be strongly annihilated by optical pumps. As for the air space distance, it is according to the numerical optimization process to realize the highest cross‐polarization conversion efficiency. We have provided the corresponding optimizing results with the spacer ranging from 20 to 60 µm, as shown in Figure S3, Supporting Information. It can be clearly visible that the broadband transmission amplitude reaches its highest value in the case of 40‐µm‐thick spacer layer. Figure 4c comparably displays the averaged *t*_yx_ as a function of pump fluence. By defining the modulation depth as *D* = (*t_yx nopump_* − *t_yx pump_*)/*t_yx nopump_* × 100%, the modulation depth can reach up to 90% with the mediation of one metallic grating. These observations unequivocally show a significant advantage in realizing THz modulations of our proposed metadevice over the conventional Fano‐type/EIT metasurfaces. Notably, the cross‐polarization conversion efficiency in our experiments is less than the corresponding theoretical data (Section S6, Supporting Information). Compared with the theoretical and simulation results, there are several factors that can decrease the efficiency in experiments. First, the Ohmic loss inherited in the gold may be higher than the ideal case that is used in our simulations (3.72 × 10^7^ S m^−1^). The higher Ohmic loss resulting from the fabrication process would undoubtedly cause the weakening of resonance strength; thus, leading to the lower conversion transmission amplitude. Second, the *z*‐cut quartz substrate cannot be clean enough after the fabrication process, especially when the spacer layer (PMMA) is spin‐coated around the metasurface. The invisible residual PMMA may cause additional loss for the resonant current in the metasurface. Third, the sizes of meta‐atoms show a slight difference between the simulated optimized ones and fabricated ones, which would result in the random shifting of resonance frequency of each meta‐atom. After adding the metallic grating, another possible reason may lead to the decrease of conversion efficiency because the efficiency can reach up to 0.5 in our simulations. The spacer distance between the metallic grating and metasurface should also be considered to improve the conversion efficiency. As for how to improve the transmission amplitude, one of the schemes has been proposed in our manuscript by using two orthogonal gratings. However, such a three‐layered structure is relatively challengeable for the fabrication. We here propose another approach with only two metallic layers to realize a relatively high value, as marked in Figure S4a, Supporting Information. Instead of the air spacer layer used in our experiments, we here replace the air spacer with one 30 µm dielectric spacer layer (quartz). The average cross‐polarized transmission can reach up to 0.65. However, the cross‐polarization conversion efficiency of such approach is also less than that of the three‐layered structure, as shown in Figure S4b, Supporting Information. Thus, the metasurface sandwiched by two orthogonal layers can be considered as a best candidate to realize the high‐efficient cross‐polarization conversion.

**Figure 5:**
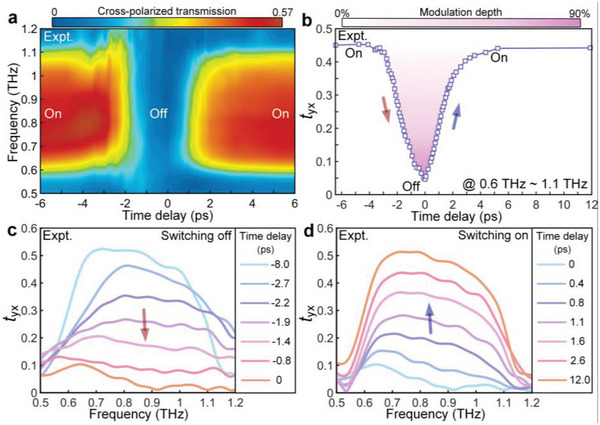
Measured transient dynamic of the cross‐polarized Fano‐type metasurface. a) Color map showing THz transmission amplitude against frequency and pump‐probe time delay over the entire ultrafast On–Off–On photo‐switching cycle. b) Averaged amplitude and normalized modulation depth of cross‐polarized transmission evaluated from the experimental results in (a). Measured THz frequency transmission spectra of the cross‐polarized Fano‐type metadevice at various time delays during the c) switching off and d) switching on periods.

A strong interaction of transient dynamic materials with Fano/EIT resonances is of vital importance in realizing ultrafast control of planar photonic metadevices. The presence of defects in semiconductors provides an additional channel to trap free carriers with faster recombination rate, while the concomitant appearance of localized energy states leads to the poor photoconductivity.^[^ ^83^, ^84^, ^85^ ^]^ Such an ultrafast free carrier relaxation is treated as a transient perturbation on the metasurface. The unique feature of swift relaxation in the 200‐nm‐thick Ge film occurs with sub‐picosecond lifetime, as shown in Figure S6, Supporting Information. Pumping the THz metadevice with an optical beam of wavelength 800 nm (1.55 eV), the dynamics of broadband THz switching can be unveiled by the optical pump and terahertz probe (OPTP) technique. The frequency‐dependent transmission spectra as a function of time delay are shown in **Figure** **5**a. It is interesting to note that the metadevice not only provides a high‐contract binary‐level switching (On–Off) in a broadband frequency range but also allows for On–Off–On switching cycle less than 10 ps. Such an excellent performance starkly contrasts with the previous EIT/Fano‐type metadevices which operate at high‐Q based narrow frequency range.^[^ ^56^, ^64^, ^65^, ^66^, ^69^, ^75^ ^]^ To further quantify the switching functionality, Figure 5b precisely tracks the time dependence of ultrafast switching process when an averaged cross‐polarized transmission ranging from 0.6 to 1.1 THz is plotted. The transmission amplitude remains unaffected before −4 ps as 0.47; and then, we clearly observe a drop to 0.047 at 0 ps. It then recovers to the original value at 5 ps and remains as a constant with no appreciable change after this time delay. Meanwhile, a 90% modulation depth within the 9‐ps switching cycle is also quantitatively identified in Figure 5b. Figure 5c,d depicts the detailed THz spectra evolutions during the switching off and switching on processes, respectively. The transmission at frequencies from 0.6 to 1.1 THz are all strongly affected by presence of free carriers, determining a highly efficient broadband ultrafast switching.

**Figure 6:**
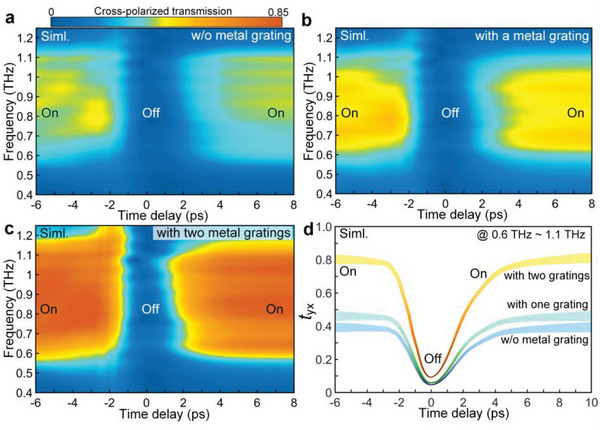
Numerical results of the ultrafast photo‐switching cross‐polarized THz wave. a–c) Simulated color maps over the entire ultrafast photo‐switching cycle showing the ultraefficient tunability of the broadband THz wave. Three types of combinations are designed to realize the maximal conversion efficiency and modulation depth: a single layer cross‐polarized Fano‐type metasurface without metallic grating(a); with one metallic grating below the metasurface(b); the metasurface sandwiched by two orthogonal metallic gratings (c). d) Averaged amplitude of cross‐polarized transmission evaluated from the numerical results in (a–c).

In addition to evaluating the broadband transmission tunability and temporal response, we also try to explore the possibility of enhancing the polarization conversion efficiency by adding metallic gratings. The measured data serves as a direct example to show that the mediation of one metallic grating can improve the broadband cross‐polarization conversion. This consideration motivates us to design a proper multilayer stack to realize broadband, ultrafast, high contrast THz transmission tuning simultaneously. To fully reproduce the transient dynamics in the experiments, the Ge film is modeled as a time‐varying material in the time‐domain solver of COMSOL Multiphysics. The conductivity of Ge is obtained from the measured data in Figure S6, Supporting Information, according to Δ*σ*(*t*) = *ε*_0_ *c*/*d*(*n_a_* + *n_s_*)[ − Δ*E*(*t*)/*E*_0_]. The transient dynamics in **Figure** **6**b are qualitatively the same with the experimental results in Figure 5a. The numerical calculations can thus constitute a testbed for searching a perfect design. The spectral evolutions are comparably demonstrated in Figure 6a–c in the cases with a single layer cross‐polarized Fano metasurface, hybridized with one metallic grating, sandwiched by two orthogonal gratings, respectively. Three configurations hold appreciate features of high modulation depth larger than 90% and fast switching cycle within 10 ps. The most striking feature; however, is the phenomenon that the broadband cross‐polarized transmission is greatly boosted exceeding 0.8 (yellow curve, Figure 6d). It implies the metadevice can complete a broadband transmission amplitude change over 0.75 with an ultrafast modulation speed over one hundred of gigahertz. It can be clearly visible in Figure 6d that the slope of the curve versus time delay is shaper when the gratings are added. As shown in Figure 6d, it is observed that the switching‐off processes all start from ≈ −2 ps and the switching‐on processes all complete at ≈4 ps. Then, we can notice that the transmission amplitudes at 0 ps for three cases are almost the same. However, their switching‐on amplitudes show a great difference (0.4, 0.5, and 0.8). As a result, the curve slopes of these cases demonstrate an obvious difference. Please note that the photoconductivity in semiconductors is strongly dependent on the lifetime of photocarriers. Typically speaking, the shorter of the photocarriers indicates a lower photoconductivity because of the appearance of defect‐recombination energy band.^[^ ^84^ ^]^ For instance, unlike the two previous works whose carriers’ lifetimes are ≈500 ps and ≈1000 ps,^[^ ^86^, ^87^ ^]^ respectively, the semiconductor used in our work holds a photocarrier's lifetime less than 1 ps. Such a short lifetime can ensure an ultrafast modulation cycle within several picoseconds but sacrificing the high photoconductivity. As reported previously, a relatively high pump fluence (1.4 mJ cm^−2^) is needed to modulate a sharp resonance even though a sensitive Fano‐type meta‐atom was used.^[^ ^65^ ^]^

The high performance of the cross‐polarized Fano metasurface can be illustrated by considering the near‐field distributions induced in the metamaterials at their respective resonant frequencies, as is shown in Figure 2. As mentioned in Figure 1c, the resonant phase difference between resonators of “m” and “n” would strongly influence the uniformity of broadband polarization conversion. Figure 2a–c validates the concept of C_2_ symmetrical arrangement of SRRs to achieve such a phase diagram. Here, the small SRRs are referred to as the resonators of “n”, while the larger ones correspond to “m”. As an *x*‐polarized THz wave impinges on the metasurface, only the CW is excited with an oscillated current density. The field profiles of both small and large SRRs show excitation of the LC mode by near‐coupling from the CW at 0.99 and 0.70 THz, respectively. Nevertheless, the two LC modes do exhibit an out‐of‐phase distribution but it does not impact on the far‐field radiation owing to the different eigenfrequencies. At the frequency of 0.86 THz, a super‐hybrid mode is formed through the mutual coupling from the dipole mode and two LC modes. Regarding the modes that radiate *y*‐polarized waves, four SRRs underpin the unique characteristic of in‐phase oscillations. The presence of such an alive mode plays a key role in determining a broadband cross‐polarization conversion uniformly.

To study the physical mechanism behind the operation of meta‐switch, we calculate the near‐field enhancements at various frequencies of the transmission spectrum (see Figure 2d–i). Instead of using a time‐varying material, the Ge film modeled in the frequency solver is approximated using an increase in its DC‐conductivity. At the eigenfrequencies of “n” and “m” (0.70 and 0.99 THz), there is strong confinement of electric fields in the capacitive split gaps of either large SRRs or small SRRs, as shown in Figure 2d,f. Looking into Figure 2e, the super‐hybrid mode field confinement within the split gaps is reduced to a certain extent, which indicates the relatively weak resonance of each LC mode. Remarkably, the number of SRRs involved is doubled from 2 to 4; thus, ensuring enough scattering power for polarization conversion. Upon applying the perturbation with the conductivity of Ge increasing from 0 to 3600 S m^−1^, they all turn into a contrasting situation that no near‐field enhancement is observed. Consequently, the cross‐polarization efficiency dramatically drops as a result of the nonradiative damping rate increase by weakly short‐circuiting split gaps. The monitored near‐field variation under conductive perturbations highlights the usefulness of the cross‐polarized Fano concept in designing active tunable metasurfaces.

It is worth stressing that this work is based on the generalized phase diagram to control Fano resonance actively with the cross‐polarized broadband conversion, which bears some distinguishing advantages when compared with previous polarization conversion works. For instance, a broadband cross polarization conversion has been realized via twofold rotational symmetry and Fabry–Perot cavity effect.^[^ ^88^, ^89^, ^90^ ^]^ However, such broadband feature is mainly based on low Q‐factor modes, which may hinder the sensitivity of surrounding perturbations. Another scheme to realize relatively high‐Q based cross‐polarized conversion is that the bright meta‐atom is inductively coupled to the dark meta‐atom with LC resonance.^[^ ^81^, ^82^ ^]^ Such a bright‐dark coupled meta‐molecule also has tremendous capacity to generate cross polarized radiation but with limited bandwidth. By revisiting a generic model, such as the TCMT, our work provides a potential route to overcome this limitation with a broadband conversion feature based on high‐Q factor resonances.

## 3 Discussion

In summary, we have proposed a novel Fano‐type metasurface with its TMTC theory approaching the ultimate ability of efficient broadband THz transmission modulation at picosecond timescale. The metasurface devices exhibited superior performance with high modulation depth, ultrafast switching speed, and broadband frequency range. We started with a derivation of a formal theoretical framework that fully describes the spectral responses, in which all physical parameters are involved in the three‐resonators coupled system containing one *x*‐polarized eigenmode and two *y*‐polarized eigenmodes. After testing the lineshape tailoring of how to realize a broadband uniform polarization conversion, we meticulously designed a photon cavity, named as achromatic Fano‐type metasurface, completely satisfying the theoretical prediction. For the application of the concept in high‐performance THz wave tailoring, an active metadevice was fabricated by hybridizing a photoactive layer into the metasurface. Benefiting from the high sensitivity of Fano‐type resonances, the cross‐polarized leaky coupled modes could be all effectively switched off. As an active tuning knob, a transient loss with sub‐picosecond lifetime was introduced by femtosecond laser pulse injections. Transmission modulation in the broadband spectral range from 0.6 to 1.1 THz was successfully realized, featuring its merits of modulation depth over 90% and On–Off–On switching cycle less than 10 ps. On the basis of the demonstrated metadevice, the broadband conversion efficiency was markedly increased when mediated by a pair of metallic gratings; thus, leading to a higher contrasting modulation depth.

Our design concept is generic and extends the physics of Fano resonances, which is easily applicable to other frequency regions and possibly suitable to dielectric metasurfaces. It can also be applied to realize a variety of optical metasurfaces with different active functions, such as broadband sensitive sensing, achromatic dynamic imaging, and multiband communications, among others. Our work will foster a profound research area, in which diversified merits are elegantly integrated in one nano/micro‐photonic device with superior performance. With the continuous drive for practical applications, this new device paradigm will certainly excite exquisite fundamental research and facilitate profound technological advancements.

## 4 Experimental Section

### Sample Fabrication

First, a conventional photolithography method is used to fabricate the metasurface and metallic grating. Positive photoresist spin‐coated on two 2‐mm‐thick *z*‐cut quartz substrates was first patterned with the coupling resonators and grating patterns using a photomask, respectively. An electron‐beam evaporative deposition was adopted to obtain a 200‐nm‐thick gold layer covering on the patterned wafer, followed by a lift‐off of the metal, leading to the formation of metallic structures. To ensure full covering of the THz spot, the array size of the metadevice was ≈10 mm × 10 mm and the THz beam just illuminated the central area of the metasurface. Second, Ge was deposited at a rate of 2 Å s^−1^ on the EIT metasurface via electron‐beam evaporation. The thickness of the non‐crystalline Ge film was 200 nm, filling the gaps of all split ring resonators. Last, a 40‐µm‐thick PMMA layer was spin‐coated on the substrate uniformly and the center area was cut out with a knife. The metasurface and metallic grating were adhered together by leaving a 40‐µm‐thick air space between them.

### Optical Pump–THz Probe Spectroscopy and Dynamic Measurements

The measurements were performed using an optical pump‐THz probe (OPTP) spectroscopy setup, where the time‐domain electric field of the THz wave could be monitored. A Ti:Sapphire amplifier laser generated femtosecond pulses with central wavelength 800 nm, repetition rate 1 kHz, and pulse width ≈100 fs. The terahertz radiation came from the illumination of a 1‐mm‐thick ZnTe crystal by the femtosecond laser. Two balanced photodiodes linked with a lock‐in amplifier were used to detect the THz time‐domain signal via electro‐optic sampling (EOS) in a ZnTe crystal. The external pump beam was incident on the sample covering a circular area ≈5 mm in diameter, guaranteeing a uniform pump of the area probed by the THz pulse (≈4 mm in diameter). To realize polarization‐resolved terahertz spectroscopy, three wire‐grid polarizers with the extinction ratio of 10 000:1 were inserted in front of the EOS crystal. The metasurfaces were illuminated at normal incidence by an *x*‐polarized THz beam. The amplitudes of transmission were defined as $t_{yx}=\left|E_{y}^{S}\left(\omega \right)/E_{x}^{Ref}\left(\omega \right)\right|$, $t_{xx}=\left|E_{x}^{S}\left(\omega \right)/E_{x}^{Ref}\left(\omega \right)\right|$ for the cross‐ and co‐ polarized transmission, respectively. Here, *E* (*ω*) represents the frequency‐resolved far‐field electric field amplitude, which was the Fourier transformation of the time‐domain THz pulse. The measurements were all carried out in a dry air atmosphere with humidity less than 2% so that the water vapor absorption was nullified.

### Electromagnetic Simulations

The numerical results were reproduced via a commercially available software COMSOL Multiphysics. In simulations, periodic boundary conditions along *x*‐ and *y*‐directions and perfect matched layer along the *z*‐direction were selected to represent the metasurface array. Frequency‐domain solver was used to obtain the transmission spectra and near‐field distributions when the photoconductivity of the Ge film was fixed as a constant. A plane wave polarized along *x*‐direction was defined as a source to mimic the performance of the active modulation of THz waves. As for the transient dynamic simulations, the conductivity variation of the Ge film was substituted into the time‐domain solver according to the formula Δ*σ*(*t*) = *ε*_0_ *c*/*d*(*n*_a_ + *n*_s_)[ − Δ*E*(*t*)/*E*_0_]. Here, − Δ*E*(*t*)/*E*_0_ was the measured THz transmission amplitude change under the optical pump on Ge film. The conductivity of the gold was modelled as a DC value of *σ* = 3.72 × 10^7^ S m^−1^ in the frequency range of interest, and *z*‐cut quartz was a lossless dielectric with a permittivity of 3.9. The Ge‐deposition layer was set with a permittivity of 16 and the conductivity value was adjustable according to the photoconductivity extracted from measurements.

## Conflict of Interest

The authors declare no conflict of interest.

## Author Contributions

Y.H. and M.T. contributed equally to this paper. This manuscript was written through the contributions of all authors. All authors have approved the final version of the manuscript.

## Supporting information

Supporting Information

## Data Availability

The data that support the findings of this study are available from the corresponding author upon reasonable request.
