# Supplementary material for: Reassessing Fano Resonance for Broadband, High‐Efficiency, and Ultrafast Terahertz Wave Switching
Source: Adv Sci (Weinh). 2022 Nov 17;10(2):2204494. doi: 10.1002/advs.202204494 (PMC9839846; doi:10.1002/advs.202204494)
Supplement: Supplementary file 1 — Supporting Information [file ADVS-10-2204494-s001.pdf]

Supporting Information

Yuze Hu[*,†,1], *Mingyu Tong*[†,2], *Siyang Hu*[2], *Weibao He*[2], *Xiang'ai Cheng*[2], *Tian Jiang*[*,1]
[1]Institute for Quantum Science and Technology, College of Science,
National University of Defense Technology, Changsha 410073, P. R. China
[2]College of Advanced Interdisciplinary Studies, National University of Defense Technology, Changsha 410073, P. R. China
Correspondence:
Professor Yuze Hu, E-mail: hyz_yj@sina.com
Professor Tian Jiang, E-mail: tjiang@nudt.edu.cn

1. Transmission formula derived from polarization resolved coupled-mode theory

Solving Equation (1-4), we can thus obtain the following expression for the cross-polarized transmission coefficients:

$$t_{yx} = s_{2y}^{-}/s_{1x}^{+} = F\left(f_i, \kappa_{ij}, \gamma_i, \gamma_i'\right) = A/(B\kappa_{pxmy}\kappa_{pxny}) \tag{S1}$$

where

$$A = d_{1\mathrm{px}}^{*}\left(\begin{array}{c} \kappa_{\mathrm{pxmy}}\, d_{2\mathrm{ny}}\, \gamma_{\mathrm{my}}\, |\kappa_{\mathrm{pxny}}|^2 - \kappa_{\mathrm{pxny}}\, X_{\mathrm{m}y\mathrm{n}y}\, d_{2\mathrm{ny}}\, |\kappa_{\mathrm{pxmy}}|^2 - \kappa_{\mathrm{pxmy}}\, X_{\mathrm{m}y\mathrm{n}y}\, d_{2\mathrm{my}}\, |\kappa_{\mathrm{pxny}}|^2 \\ + \kappa_{\mathrm{pxny}}\, d_{2\mathrm{my}}\, \gamma_{\mathrm{ny}}\, |\kappa_{\mathrm{pxmy}}|^2 + \kappa_{\mathrm{pxmy}}\, d_{2\mathrm{ny}}\, \gamma'_{\mathrm{my}}\, |\kappa_{\mathrm{pxny}}|^2 + \kappa_{\mathrm{pxny}}\, d_{2\mathrm{my}}\, \gamma'_{\mathrm{ny}}\, |\kappa_{\mathrm{pxmy}}|^2 \\ -i\kappa_{\mathrm{pxny}}\, d_{2\mathrm{my}}\, f\, |\kappa_{\mathrm{pxmy}}|^2 - i\kappa_{\mathrm{pxmy}}\, d_{2\mathrm{ny}}\, f\, |\kappa_{\mathrm{pxny}}|^2 + i\kappa_{\mathrm{pxmy}}\, d_{2\mathrm{ny}}\, f_{\mathrm{my}}\, |\kappa_{\mathrm{pxny}}|^2 \\ + i\kappa_{\mathrm{pxny}}\, d_{2\mathrm{my}}\, f_{\mathrm{ny}}\, |\kappa_{\mathrm{pxmy}}|^2 \end{array}\right)$$

$$\begin{aligned} B = {} & X_{\mathrm{m}y\mathrm{n}y}^2 f_{\mathrm{px}} - X_{\mathrm{m}y\mathrm{n}y}^2 f - iX_{\mathrm{m}y\mathrm{n}y}^2 \gamma_{\mathrm{px}} - iX_{\mathrm{m}y\mathrm{n}y}^2 \gamma'_{\mathrm{px}} + f^2 f_{\mathrm{my}} + f^2 f_{\mathrm{ny}} + f^2 f_{\mathrm{px}} - if^2 \gamma_{\mathrm{my}} - if^2 \gamma'_{\mathrm{my}} \\ & - if^2 \gamma_{\mathrm{ny}} - if^2 \gamma'_{\mathrm{ny}} - if^2 \gamma_{\mathrm{px}} - if^2 \gamma'_{\mathrm{px}} - f^3 + f\, |\kappa_{\mathrm{pxmy}}|^2 + f\, |\kappa_{\mathrm{pxny}}|^2 - f_{\mathrm{my}}\, |\kappa_{\mathrm{pxny}}|^2 - f_{\mathrm{ny}}\, |\kappa_{\mathrm{pxmy}}|^2 \\ & + i\gamma_{\mathrm{my}}\, |\kappa_{\mathrm{pxny}}|^2 + i\gamma_{\mathrm{ny}}\, |\kappa_{\mathrm{pxmy}}|^2 + i\gamma'_{\mathrm{my}}\, |\kappa_{\mathrm{pxny}}|^2 + i\gamma'_{\mathrm{ny}}\, |\kappa_{\mathrm{pxmy}}|^2 - ff_{\mathrm{my}} f_{\mathrm{ny}} - ff_{\mathrm{my}} f_{\mathrm{px}} - ff_{\mathrm{ny}} f_{\mathrm{px}} \\ & + f_{\mathrm{my}} f_{\mathrm{ny}} f_{\mathrm{px}} + iff_{\mathrm{my}} \gamma_{\mathrm{ny}} + iff_{\mathrm{my}} \gamma'_{\mathrm{ny}} + iff_{\mathrm{my}} \gamma_{\mathrm{px}} + iff_{\mathrm{my}} \gamma'_{\mathrm{px}} + iff_{\mathrm{ny}} \gamma_{\mathrm{my}} + iff_{\mathrm{ny}} \gamma'_{\mathrm{my}} + iff_{\mathrm{ny}} \gamma_{\mathrm{px}} \\ & + iff_{\mathrm{ny}} \gamma'_{\mathrm{px}} + iff_{\mathrm{px}} \gamma_{\mathrm{my}} + iff_{\mathrm{px}} \gamma'_{\mathrm{my}} + iff_{\mathrm{px}} \gamma_{\mathrm{ny}} + iff_{\mathrm{px}} \gamma'_{\mathrm{ny}} - if_{\mathrm{my}} f_{\mathrm{ny}} \gamma_{\mathrm{px}} - if_{\mathrm{my}} f_{\mathrm{ny}} \gamma'_{\mathrm{px}} - if_{\mathrm{my}} f_{\mathrm{px}} \gamma_{\mathrm{ny}} \\ & - if_{\mathrm{my}} f_{\mathrm{px}} \gamma'_{\mathrm{ny}} - if_{\mathrm{ny}} f_{\mathrm{px}} \gamma_{\mathrm{my}} - if_{\mathrm{ny}} f_{\mathrm{px}} \gamma'_{\mathrm{my}} + f\gamma_{\mathrm{my}} \gamma_{\mathrm{ny}} + f\gamma_{\mathrm{my}} \gamma'_{\mathrm{ny}} + f\gamma_{\mathrm{my}} \gamma_{\mathrm{px}} + f\gamma'_{\mathrm{my}} \gamma_{\mathrm{ny}} \\ & + f\gamma_{\mathrm{my}} \gamma'_{\mathrm{px}} + f\gamma'_{\mathrm{my}} \gamma'_{\mathrm{ny}} + f\gamma'_{\mathrm{my}} \gamma_{\mathrm{px}} + f\gamma_{\mathrm{ny}} \gamma_{\mathrm{px}} + f\gamma'_{\mathrm{my}} \gamma'_{\mathrm{px}} + f\gamma_{\mathrm{ny}} \gamma'_{\mathrm{px}} + f\gamma'_{\mathrm{ny}} \gamma_{\mathrm{px}} + f\gamma'_{\mathrm{ny}} \gamma'_{\mathrm{px}} \\ & - f_{\mathrm{my}} \gamma_{\mathrm{ny}} \gamma_{\mathrm{px}} - f_{\mathrm{my}} \gamma_{\mathrm{ny}} \gamma'_{\mathrm{px}} - f_{\mathrm{my}} \gamma'_{\mathrm{ny}} \gamma_{\mathrm{px}} - f_{\mathrm{my}} \gamma'_{\mathrm{ny}} \gamma'_{\mathrm{px}} - f_{\mathrm{ny}} \gamma_{\mathrm{my}} \gamma_{\mathrm{px}} - f_{\mathrm{ny}} \gamma_{\mathrm{my}} \gamma'_{\mathrm{px}} - f_{\mathrm{ny}} \gamma'_{\mathrm{my}} \gamma_{\mathrm{px}} \\ & - f_{\mathrm{ny}} \gamma'_{\mathrm{my}} \gamma'_{\mathrm{px}} - f_{\mathrm{px}} \gamma_{\mathrm{my}} \gamma_{\mathrm{ny}} - f_{\mathrm{px}} \gamma_{\mathrm{my}} \gamma'_{\mathrm{ny}} - f_{\mathrm{px}} \gamma'_{\mathrm{my}} \gamma_{\mathrm{ny}} - f_{\mathrm{px}} \gamma'_{\mathrm{my}} \gamma'_{\mathrm{ny}} + i\gamma_{\mathrm{my}} \gamma_{\mathrm{ny}} \gamma_{\mathrm{px}} + i\gamma_{\mathrm{my}} \gamma_{\mathrm{ny}} \gamma'_{\mathrm{px}} \\ & + i\gamma_{\mathrm{my}} \gamma'_{\mathrm{ny}} \gamma_{\mathrm{px}} + i\gamma'_{\mathrm{my}} \gamma_{\mathrm{ny}} \gamma_{\mathrm{px}} + i\gamma_{\mathrm{my}} \gamma'_{\mathrm{ny}} \gamma'_{\mathrm{px}} + i\gamma'_{\mathrm{my}} \gamma_{\mathrm{ny}} \gamma'_{\mathrm{px}} + i\gamma'_{\mathrm{my}} \gamma'_{\mathrm{ny}} \gamma_{\mathrm{px}} + i\gamma'_{\mathrm{my}} \gamma'_{\mathrm{ny}} \gamma'_{\mathrm{px}} \\ & - \frac{i\kappa_{\mathrm{pxny}}\, X_{\mathrm{m}y\mathrm{n}y}\, |\kappa_{\mathrm{pxmy}}|^2}{\kappa_{\mathrm{pxmy}}} - \frac{i\kappa_{\mathrm{pxmy}}\, X_{\mathrm{m}y\mathrm{n}y}\, |\kappa_{\mathrm{pxny}}|^2}{\kappa_{\mathrm{pxny}}} \end{aligned}$$

According to the energy conservation law and time-reversal symmetry, we can obtain:

$$d_{1px} = i\sqrt{2}\sqrt{\frac{\gamma_{px}}{\eta^2+1}}$$

$$d_{2px} = i\sqrt{2}\,\eta\sqrt{\frac{\gamma_{px}}{\eta^2+1}}$$

$$d_{1my} = i\sqrt{2}\sqrt{\frac{\gamma_{my}}{\eta^2+1}}$$

$$d_{2my} = i\sqrt{2}\,\eta\sqrt{\frac{\gamma_{my}}{\eta^2+1}}$$

$$d_{1ny} = i\sqrt{2}\sqrt{\frac{\gamma_{ny}}{\eta^2+1}}$$

$$d_{2ny} = i\sqrt{2}\,\eta\sqrt{\frac{\gamma_{ny}}{\eta^2+1}}$$

$$X_{myny} = 2\sqrt{\gamma_{my}\gamma_{ny}}/(\eta^2+1)$$

where $\eta = \sqrt{n_{sub}}$ represents the background asymmetry transmisssion from two ports caused by the presence of a substrate used in our experiment.

2. Multipole decomposition

The optical modes that contribute to each multipole moment in the far-field are analysed using the multipolar decomposition method. These physical values could be expressed using the Cartesian basis with numerical integration of the current density inside a single unit cell volume V as follows:

$$\mathbf{P} = \frac{1}{i\omega}\iiint_V \mathbf{J}\,dr^3$$

$$\mathbf{T} = \frac{1}{10c}\iiint_V [(\mathbf{r}\cdot\mathbf{J})\mathbf{r} - 2\mathbf{r}^2\mathbf{J}]\,dr^3$$

$$\mathbf{M} = \frac{1}{2c}\iiint_V (\mathbf{r}\times\mathbf{J})\,dr^3$$

$$\mathbf{Q}^{(\mathbf{e})}_{\alpha,\beta} = \frac{1}{2i\omega}\iiint_V \left[r_\alpha J_\beta + r_\beta J_\alpha - \frac{2}{3}\delta_{\alpha,\beta}(\mathbf{r}\cdot\mathbf{J})\right]dr^3$$

$$\mathbf{Q}^{(\mathbf{m})}_{\alpha,\beta} = \frac{1}{3c}\iiint_V [(\mathbf{r}\times\mathbf{J})_\alpha r_\beta + (\mathbf{r}\times\mathbf{J})_\beta r_\alpha]\,dr^3$$

where $\boldsymbol{r}$ is the position vector, c is the speed of light in vacuum, and $\boldsymbol{J}$ is the current density. Here, the software of COMSOL Multiphysics is used as a tool to carry out the multipole decomposition. The multipole moments associated distributed powers are as follows:

$$I_{\mathbf{P}} = \frac{2\omega^4}{3c^3}|\mathbf{P}|^2$$

$$I_{\mathbf{T}} = \frac{2\omega^6}{3c^5}|\mathbf{T}|^2$$

$$I_{\mathbf{M}} = \frac{2\omega^4}{3c^3}|\mathbf{M}|^2$$

$$I_{\mathbf{Q}^{(\mathbf{e})}} = \frac{\omega^6}{5c^5}|\mathbf{Q}^{(\mathbf{e})}_{\alpha,\beta}|^2$$

$$I_{\mathbf{Q}^{(\mathbf{m})}} = \frac{\omega^6}{40c^5}|\mathbf{Q}^{(\mathbf{m})}_{\alpha,\beta}|^2$$

3. Scattering powers and transmissions with different meta-atom configurations

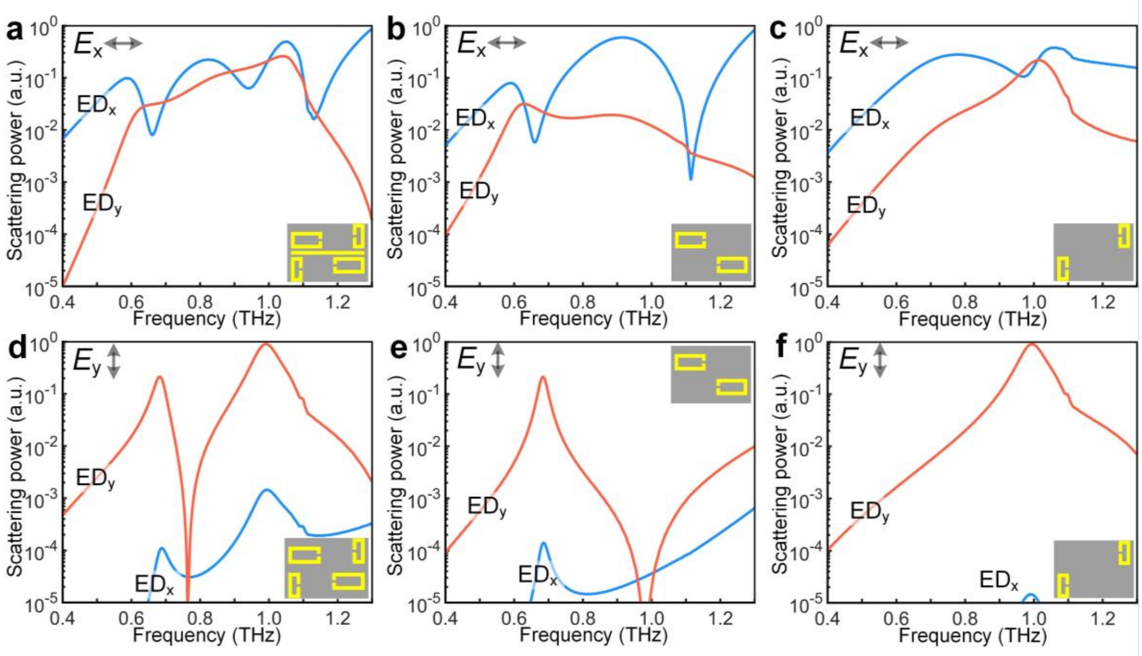

Figure S1 Far-field scattering power of two electric dipoles for different metaatom configurations. Scattering power excited by the (a-c) x-polarized THz incidence and (d-f) y-polarized THz incidence. The insets showing the top-views of the corresponding metaatom configuration.

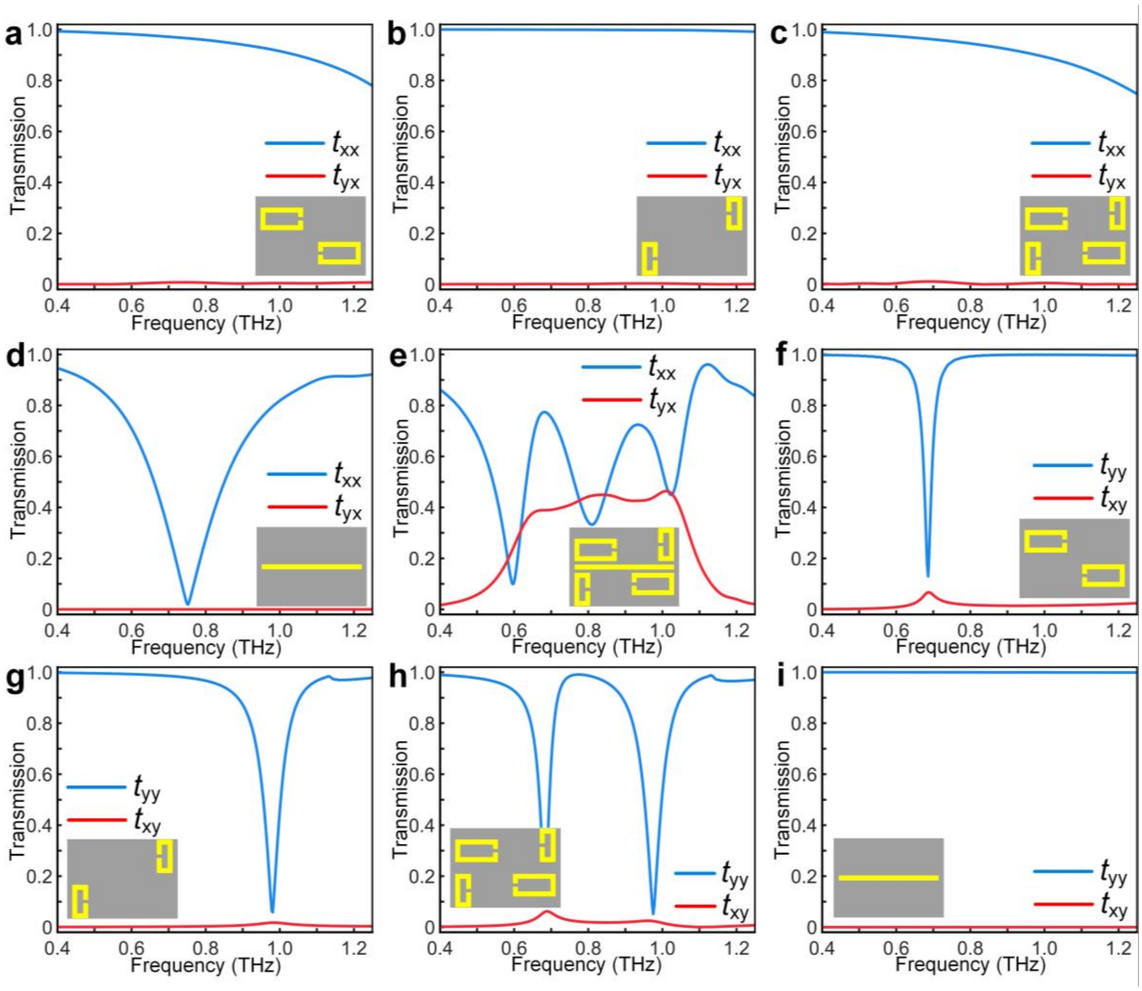

Figure S2 Co- and cross-polarized THz transmission spectra for different metaatom configurations. (a-e) Excited by x-polarized THz incidence and (f-i) excited by y-polarized THz incidence. The insets showing the top-views of the corresponding metaatom configuration.

4. Optimizing processing for the air space distance between the metasurface and metallic grating

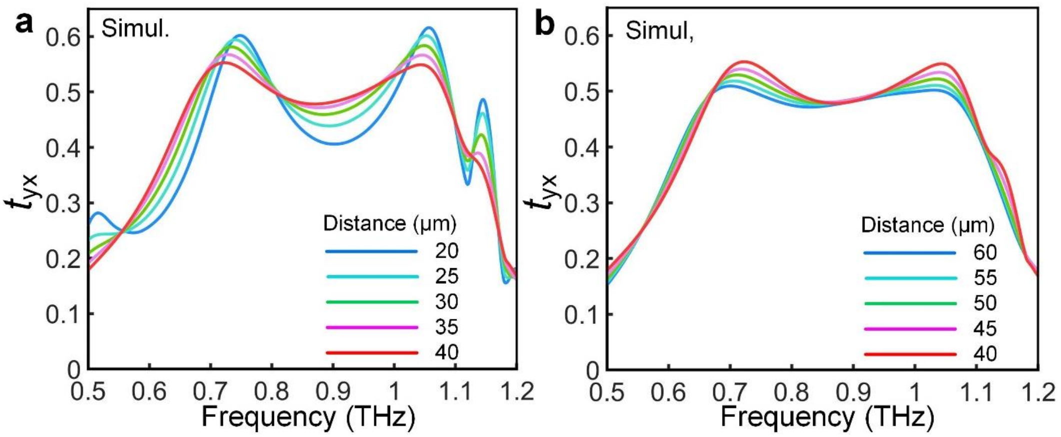

Figure S3. The cross-polarized transmission amplitude as a function of the spacer distance between the metasurface and the metallic grating layer.

5. Two schemes to improve the polarization conversion efficiency

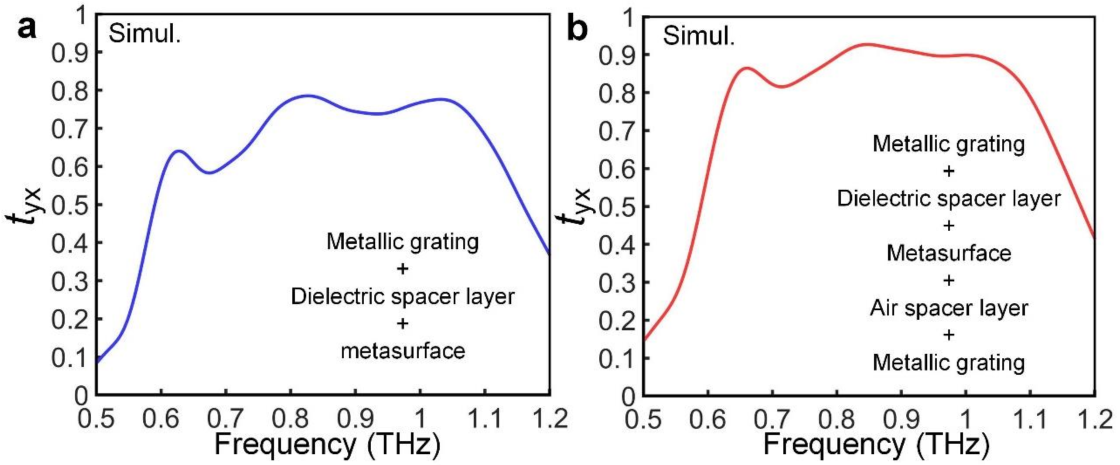

Figure S4. Two ways to improve cross-conversion efficiency. (a) Two-layered structure with a dielectric spacer layer. (b) Three-layered structure with a metasurface sandwiched by two orthogonal metallic gratings.

6. Comparison between the experimental data and the simulation results

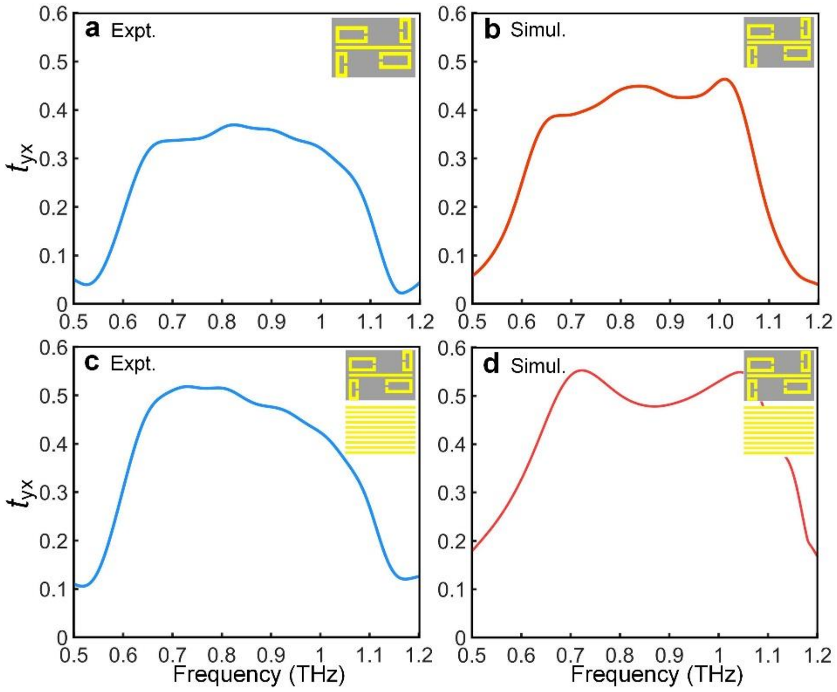

Figure S5. The comparison of cross-polarized transmission between the numerical simulations and the experiments. (a)-(b) The cross-polarized transmissions of a single layer metasurface. (c)-(d) The cross-polarized transmissions of a bilayer structure with an air spacer.

7. OPTP measurements for an amorphous Ge film

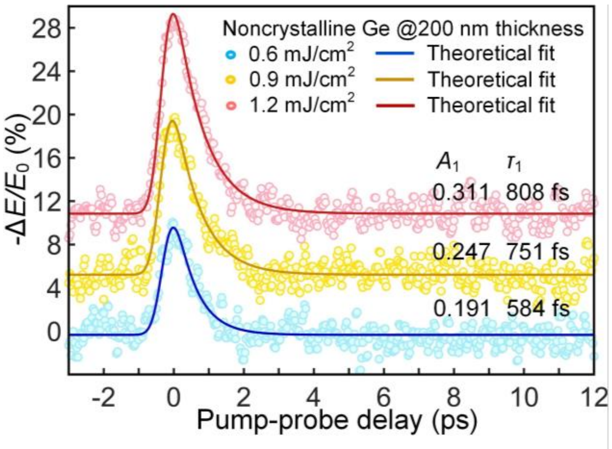

Figure S6. Sub-picosecond time scale photocarrier dynamics of the amorphous Ge film. Dots representing measured THz transmission amplitude change under the optical pump with different pump fluences. Solid curves showing the theoretically fittings.

8. Comparison between different active metamaterial schemes

Table S1.

Trigger type	Active media /techniques	Time scale	Amplitude modulation depth	Mode	Modulation frequency range	Year
Optical	Silicon on sapphire[1]	~ns	26%	Fano	0.6 THz – 0.7 THz	2017
Optical	Germanium & Silicon on sapphire[2]	~ps to ~ns	25%	EIT	0.65 THz – 0.7 THz	2020
Optical	Germanium on kapton[3]	~ps	21%	Fano	0.85 THz – 0.95 THz	2018
Optical / Electrical	Chalcogenide Phase Change Material[4]	~ps / ~s	27%	Fano	0.8 THz – 0.9 THz	2019
Optical	40 nm-thick WSe_2[5]	~ps	30%	PIT	0.75 THz – 0.81 THz	2020
Optical	Superconducto r (YBCO)[6]	~ps	50%	Dipole	0.4 THz – 0.5 THz	2012
Optical	Hybrid Lead Halide Perovskites[7]	~ sub-ns	18%	Fano	0.77 THz – 0.83 THz	2017
Optical	Silicon on sapphire[8]	~ns	40%	Toroidal	0.52 THz – 0.6 THz	2018
Electrical	Graphene–ferroelectric[9]	~s	10%	Dipole	1.0 THz – 1.3 THz	2016
Electrical	VO_2[10]	~s	57%	Dipole	0.55 THz – 0.75 THz	2018
Thermal	VO_2[11]	~s	75%	Dipole	0.8 THz – 1 THz	2022
Optical	Germanium	**~ps**	**45% & 72%**	Fano	**0.6 THz - 1.1 THz**	Our work

[1] M. Manjappa, Y. K. Srivastava, L. Cong, I. Al-Naib, R. Singh, Adv. Mater. 2017, 29, 1603355.

[2] Y. Hu, J. You, M. Tong, X. Zheng, Z. Xu, X. Cheng, T. Jiang, Advanced Science 2020, 7, 2000799.

[3] W. X. Lim, M. Manjappa, Y. K. Srivastava, L. Cong, A. Kumar, K. F. MacDonald, R. Singh, Adv. Mater. 2018, 30, 1705331.

[4] P. Pitchappa, A. Kumar, S. Prakash, H. Jani, T. Venkatesan, R. Singh, Adv. Mater. 2019, 31, 1808157.

[5] Y. Hu, T. Jiang, J. Zhou, H. Hao, H. Sun, H. Ouyang, M. Tong, Y. Tang, H. Li, J. You, X. Zheng, Z. Xu, X. Cheng, Nano Energy 2020, 68, 104280.

[6] R. Singh, J. Xiong, A. K. Azad, H. Yang, S. A. Trugman, Q. X. Jia, A. J. Taylor, H.-T. Chen, Nanophotonics 2012, 1, 117.

[7] M. Manjappa, Y. K. Srivastava, A. Solanki, A. Kumar, T. C. Sum, R. Singh, Adv. Mater. 2017, 29, 1605881.

[8] M. Gupta, Y. K. Srivastava, R. Singh, Adv. Mater. 2018, 30, 1704845.

[9] W. Y. Kim, H.-D. Kim, T.-T. Kim, H.-S. Park, K. Lee, H. J. Choi, S. H. Lee, J. Son, N. Park, B. Min, Nat. Commun. 2016, 7, 10429.

[10] H. Cai, S. Chen, C. Zou, Q. Huang, Y. Liu, X. Hu, Z. Fu, Y. Zhao, H. He, Y. Lu, Adv. Opt. Mater. 2018, 6, 1800257.

[11] X. Zhao, J. Lou, X. Xu, Y. Yu, G. Wang, J. Qi, L. Zeng, J. He, J. Liang, Y. Huang, D. Zhang, C. Chang, Adv. Opt. Mater. 2022, 2102589.
